# Effect of Increased Salt Water Intake on the Production and Composition of Dairy Goat Milk

**DOI:** 10.3390/ani11092642

**Published:** 2021-09-09

**Authors:** Roberto Germano Costa, Rayssa M. Bezerril Freire, Gherman Garcia Leal de Araújo, Rita de Cássia Ramos do Egypto Queiroga, Gutemberg Nascimento Paiva, Neila Lidiany Ribeiro, Ronaldo Lopes de Oliveira, Rubén Domínguez, José M. Lorenzo

**Affiliations:** 1 Programa de Doutorado Integrado em Zootecnia, Universidade Federal da Paraíba (UFPB), Areia 58397-000, Brazil; betogermano@hotmail.com (R.G.C.); rayssa_bezerril@yahoo.com.br (R.M.B.F.); gherman.araujo@embrapa.br (G.G.L.d.A.); rcqueiroga@uol.com.br (R.d.C.R.d.E.Q.); aviaap@hotmail.com (G.N.P.); 2 Bolsista PCI/CNPq, Instituto Nacional do Semiárido—INSA, Campina Grande 58434-700, Brazil; neilalr@hotmail.com; 3 Departamento de Zootecnia, Universidade Federal da Bahia (UFBA), Salvador 40110-909, Brazil; ronaldooliveira@ufba.br; 4 Centro Tecnológico de la Carne de Galicia, Rúa Galicia Nº 4, Parque Tecnológico de Galicia, San Cibrao das Viñas, 32900 Ourense, Spain; rubendominguez@ceteca.net; 5 Área de Tecnología de los Alimentos, Facultad de Ciencias de Ourense, Universidad de Vigo, 32004 Ourense, Spain

**Keywords:** conductivity, milk composition, physicochemical, saline water, milk quality, animal management

## Abstract

**Simple Summary:**

The high consumption of water by livestock is a major drawback from an environmental point of view. In addition, the fact that arid or semi-arid areas have water with high salinity can decrease the quality of the products derived from these animals. Therefore, the present work is proposed with the objective of understanding how the supply of water with different salinities can affect the quality of milk. Therefore, the results of this work will derive important conclusions of vital importance for farmers in these areas.

**Abstract:**

Due to its necessity and magnitude, water is essential for animal nutrition. This study aimed to evaluate the effects of increasing levels of water salinity on the quality of goat milk in the Brazilian semiarid region. Twenty-four multiparous Alpine goats, with an average live weight of 38.0 ± 4.0 kg and an average lactation period of 30 days, distributed entirely at random, were used. The experiment lasted 64 days including an initial period of 14 days of adaption to the diet. The experimental treatments consisted of water with different levels of total dissolved solids (TDS): 640, 3188, 5740, and 8326 mg L^−1^, obtained using sodium chloride (NaCl). Increasing the levels of TDS in drinking water from 640 to 8326 mg L^−1^ did not significantly (*p* > 0.05) affect the production and the physicochemical composition of the milk. There was a linear increase (*p* < 0.05) in the water consumption and acidity variables as a function of the total dissolved solid levels. The mineral composition of the milk was not altered with increasing levels of TDS in water from 640 to 8326 mg L^−1^. There was no negative effect (*p* > 0.05) for any of the sensorial attributes analyzed in relation to the treatments. Therefore, as a general conclusion, based on the analyses carried out in this experiment, it was found that water with total dissolved solids, when supplied for short periods of up to 48 days, does not alter the production, physicochemical characteristics, or the organoleptic properties of goat’s milk.

## 1. Introduction

Due to its necessity and magnitude, water is essential for animal nutrition and is indispensable to all biochemical and physiological processes of an organism, as it is the largest component of the body. Water is the component with the highest recycling rate, acting as a vehicle for nutrients in digestion, absorption, and excretion [1].

Water scarcity coupled with poor water quality in semi-arid regions are the limiting factors for the development of rural populations, as they often introduce considerable amounts of total dissolved solids (TDS) [2], making water unfit for human consumption. Since this water can be used for animal consumption, it is necessary to know the maximum concentration of salt that animals can tolerate. The quality of water ingested by animals is important to their health and their corporal and productive development because this liquid can serve as an important conveyor of physical, chemical, and microbiological contaminants [3].

Groundwater found in the semi-arid region of Northeast Brazil contains different concentrations ranging from 0.0128 mg L^−1^ TDS to 48,000 mg L^−1^ TDS [4] of good quality, electrically conductive minerals. Water with high electrical conductivity is not used for human consumption without previous treatment; in this way, the water sources for animals are increased in periods of long droughts and water shortage. Water with an electrical conductivity above 16 dS m^−1^ should not be supplied to animals [5], including ruminants, which are more tolerant to the consumption of this type of water.

Fresh water for animals must contain 3188 to 5740 mg L^−1^ TDS to improve the concentration, vigor, motility and volume of semen and to reduce sperm abnormalities. Water with high salinity (8326 mg L^−1^ TDS) should be avoided because it increases apoptosis in germinal cells of seminiferous tubules in Morada Nova lambs [6]. Understanding the physiological mechanisms underlying the ability of animals to adapt to higher ingested salt loads is a necessary step in developing sustainable strategies for raising small ruminants on saline soils [7].

The dairy goat is actively bred in the arid and semi-arid regions of the world. Goats are notable for producing foods of high nutritional value under adverse climatic and environmental conditions, a characteristic attributed to their ability to adapt to these conditions, as well as for contributing to the socioeconomic development of the populations of these regions [8]. The goat is one of the most efficient domestic animals in terms of its use of water, being able to conserve it by reducing losses in the urine and feces [1].

Goat milk is recognized worldwide because of its unique nutritional and hypoallergenic qualities [9]. The origin of the water and the conditions in which it circulates, such as the quality of the land, plumbing and reservoirs, as well as the places where it is consumed, influence the quality of the water and, consequently, the quality of the milk. In addition to these factors, the level of salinity or the content of toxic elements in the water may make milk unfit for consumption [10].

Animals can be adapted to drink saline water; however, a gradual addition is recommended because the abrupt change can result in negative influences on feed and water intake. These animals can ingest saline water with levels up to 9500 mg TDS/L with no effects on feed intake; however, higher levels decrease feed intake [11]. The time needed for goats to adapt to water with high levels of salt is unknown. However, it is fundamental to know the salinity tolerance of dairy goats raised in semi-arid regions for better use of saline waters without causing any major damage to milk production and composition. Considering the low availability of drinking water and the lack of studies related to the influence of water quality on the qualitative attributes of goat milk, this study aimed to evaluate the effects of supplying dairy goats with water with increasing levels of salt on the physicochemical characteristics and organoleptic properties of goat’s milk in the Brazilian semi-arid region.

## 2. Materials and Methods

### 2.1. Animal Diet and Management

The experiment was conducted at the Laboratory of Goat Rearing, Research Center for Human, Social, and Agricultural Sciences, Federal University of Paraíba, at Campus III, located in Bananeiras—PB, Brazil. The study was analyzed and approved by the Committee on Ethics in the Study and Research of Deontology of the Federal University of Vale do São Francisco (case no. 0007/131014).

Twenty-four multiparous Alpine goats were used, with an average live weight of 38 ± 4 kg and an average lactation of 30 days. These animals were distributed entirely at random. The experimental duration was a 14-day introduction followed by three periods of 16 days giving a total duration of 64 days. The goats were kept in an intensive system and allocated individual cages with 1.26 m^2^ of space equipped with troughs and water fountains and with floors made of railed wood crates.

The ingredients used for the formulation of the diet had the following composition: buffel grass hay (*Cenchrus ciliaris* L.) and a base made of soybean meal, corn bran and mineral 50:50 (concentration:volume) (Table 1). The experimental rations were formulated to meet the milk production requirements of 2 kg/day [12]. The ration was available in two daily servings after milking at 7:00 and 15:00 h. The dry matter intake was controlled according to the diet provided and the leftovers, which were adjusted daily to allow 20% of leftovers.

The treatments corresponded to increasing levels of total dissolved solids in the water provided to the animals, which was created using sodium chloride (NaCl), distributed into four electrical conductivity levels: 1.0, 5.0, 9.0, and 13.0 dS m^‒1^. The conductivity and temperature of each treatment were read daily using a conductivity meter (Digimed, São Paulo, Brazil) with an allowance of a difference of 5% in the limit of each treatment. To convert the electrical conductivity of the water to parts per million (ppm), or milligrams per liter of total dissolved solids (mg L^−1^ TDS), we multiplied 1 dS m^−1^ by 640 mg L^−1^ [15]. The treatments were converted to milligrams per liter in the following proportions: T1—640, T2—3188, T3—5740, and T4—8326 mg L^‒1^ TDS (Table 2). Water troughs were washed once weekly to prevent NaCl accumulating on the borders, which might affect the salt concentration of each treatment.

During the entire experiment, samples of water from each treatment were collected weekly, conditioned in labeled plastic bags and subsequently frozen until they were analyzed. The samples were sent to the Geo-Environmental Laboratory of Embrapa Semiarid, where chemical analyses for bicarbonate, chlorides, calcium, magnesium, potassium, and sodium were conducted and where the electrical conductivity of the collected water samples was also measured.

Sodium and potassium analyses were performed using the flame photometry method, where the water was diluted 0, 10, 100, and 1000 times, and each sample had four replicates; then, each sample was read after removal of all impurities. The equipment was washed with distilled water. For titration of the chlorides, the Mohr method, which relies on the titration of the water sample with silver nitrate using potassium chromate as the endpoint indicator, was used. To titrate the carbonates and bicarbonates, the volumetric or titrimetric alkaline method was used, using sulfuric acid to determine the levels of carbonates and bicarbonates and the indicators phenolphthalein and methyl orange to titrate the carbonates and the bicarbonates, respectively [16]. The calcium and magnesium analyses were performed through complexometric titration, i.e., by using EDTA (Ethylenediaminetetraacetic acid) to complex calcium and magnesium at alkaline pH levels. For the analysis of calcium, murexide was used as an indicator after the sum of the Ca + Mg was determined using Eriochrome Black T, and the Mg concentration was determined by the difference [17].

### 2.2. Milk Sampling and Analysis

For the sampling, the udders of the animals were sanitized before the milking. The milk samples were collected in three distinct 16-day periods, with collections taken on the 15th and 16th day of each experimental period by manual milking carried out at 6:00 and 14:00 h in the milking room. Aliquots of each animal’s milk were obtained as compound samples proportional to the milk production for each milking shift (70% of the milk collected in the morning and 30% in the afternoon). Polyethylene bottles of 300 mL were used for the physicochemical analysis, and 100 mL bottles were used for the analysis of minerals; the milk samples were kept under freezing temperatures (−18 °C) for further analysis.

The physicochemical analyses were performed at the Laboratory of Bromatology of the Health Sciences Center of the UFPB at the end of the experimental period. The levels of density, acidity, protein, lipids, lactose and ash were determined according to the methodology described by the AOAC [18]. For moisture, the percentage of reduction of the total dry extract (TDE) was calculated, and the defatted dry extract (DDE) was determined by calculating the TDE minus the percentage of fat [18].

The mineral analyses were carried out at the Laboratory of Chemical Analysis of Food of the UFPB. For the analysis of phosphorus and chlorides, the ash solutions were extracted and then stored in 100 mL glass pots. The phosphorus analysis was obtained by the colorimetric method, which is based on the reaction between the phosphorus of the mineral solution and ammonium molybdate, producing ammonium phosphomolybdate. The amount of phosphorus was determined by measuring the intensity of the blue color that is produced by the formation of the phosphomolybdate. For this analysis, a spectrophotometer (Coleman, model 33D, Santo André, São Paulo, Brazil) with a wavelength of 650 nm was used [19]. For the titration of chlorides, the Mohr method was used [18]. This test was based on the precipitation of chlorides in the form of silver chloride at pH 8.3 and in the presence of potassium chromate as an indicator. The end of the reaction was indicated by the formation of a brick-red precipitate of potassium chromate, and the concentration of chloride and sodium present in the sample was obtained through the following equation:

(1) $$Chloride\;\%\;\left(NaCl\right)=\frac{100xVolume\;AgNO^{3}xfx0.00585}{sample\;weight}$$

The determination of calcium was conducted by the titrimetric method based on the complexation reaction of EDTA and calcium. The potassium content was obtained by flame photometry using the MERCK^®^ brand potassium standard and a flame photometer (Tecnow 7000, São Paulo, Brazil) [18].

### 2.3. Sensory Analysis

For sensory analysis, samples of milk were collected from each treatment, and five sub-samples were prepared of one liter each. The milk was pasteurized, and sensorial analysis was made two days after cold storage. The sensory analysis was performed in individual booths in controlled environmental conditions at a temperature of around 23 °C [20].

The sensory evaluation was carried out with an internal panel consisting of 20 evaluators (aged 20 to 40 years), and 11 evaluators were selected and trained according to the methodology of Noronha [21]. Said subjects were selected for their sensory ability and trained for descriptive analysis according to the standard flavor profile guidelines set by ISO 6564:1985. Panel training sessions were performed to familiarize the assessors with the language and goat milk. The samples were described using the Quantitative Descriptive Analysis (QDA) technique [20]. The QDA test was administered using a five-point scale ranging from 1 (little) to 5 (very much) regarding the following attributes: external odor aspects (overall intensity, goat’s milk odor (butter/rancid and aromatic)), aftertaste (overall intensity and persistence), overall acceptability (disliked very much or liked very much), and flavor (overall intensity, butter/rancid, goat, aromatic) (Table 3).

The three testing sessions (trained panel and consumer testing) were conducted in individual booths under conditions in accordance with ISO 8589 (facilities) and ISO11037 (lighting). Each assessor was served four samples (each pot coming from a treatment) coded with three-digit random numbers, served immediately after being taken out of refrigerated storage with lids containing 50 mL aliquots of the duly pasteurized milk. Assessors were asked to use low-salt crackers and water to clean their palates between the assessed samples.

### 2.4. Statistical Analysis

The results were subjected to regression analysis. The sensory analyses were carried out in triplicate. The means of the results were evaluated using analysis of variance (ANOVA) and orthogonal contrast (control versus salinity water levels) (*p* < 0.05) on SAS 9.0 software. To identify the sensorial attributes that most contributed to the discrimination of the differences between goats’ milk samples submitted to treatments with increasing levels of total dissolved solids during the 48-day exposure period, a principal component analysis (PCA) was applied using the panelists’ averages.

## 3. Results

The increase in the level of TDS in fresh water from 640 to 8326 mg L^−1^ significantly affected (*p* < 0.05) the production and physicochemical composition of milk (Table 4). Water consumption and milk acidity showed a linear regression trend as levels of TDS increased, and the values of these two variables decreased.

The mineral composition of goat’s milk subjected to increasing levels of TDS had no significant effect (*p* > 0.05). The minerals phosphorus, chlorine, calcium, sodium, and potassium presented mean values of 105.7, 250.4, 129.2, 161.7, and 235.2, respectively. The milk mineral composition showed no regression tendency (Table 5).

The sensorial attributes analyzed did not present a regression tendency in relation to the treatments (Table 6), which reinforces the results observed in Table 4, indicating that the production and chemical composition of the milk did not vary depending on the total solids levels in the water.

**Figure 1:**
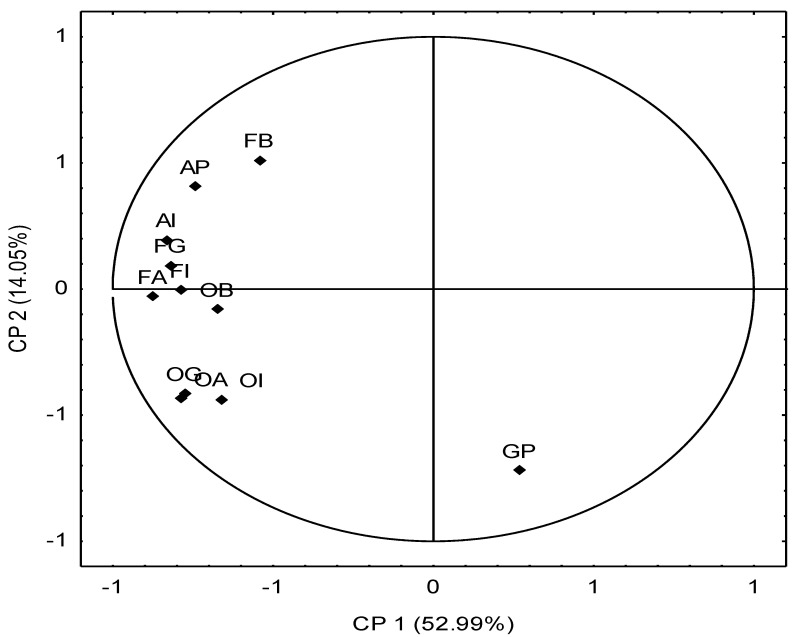
Principal component analysis of milk from goats of the Alpine breed subjected to treatments with increasing levels of total dissolved solids over a period of 48 days of exposure. Legend: FB = butter/rancid flavor; AP = aftertaste persistence; AI = aftertaste intensity; FG = goat flavor; FI = overall flavor intensity; FA = aromatic flavor; OB = butter/rancid odor; OG = goat odor; OA = aromatic odor; OI = overall odor intensity; GP = global persistence.

The trend towards the separation of variables was observed when an analysis of the main components was performed. The first two main components explained 67.04% of the data variance, and the first component was responsible for explaining 52.99% of the data variance (Figure 1).

The results of the sensorial analysis are described and represented as vectors, which correspond to the tasters and characterize the attributes that are located close to them. When evaluating the matrix, three different behaviors were observed: an isolated variable and two clusters, each forming variables that followed the same trends. A group was formed by attributes that had the same trend: overall intensity of flavor (FI), aromatic flavor (FA), aftertaste persistence (AP) and aftertaste intensity (AI); however, these attributes were in the negative quadrant, indicating that this group obtained lower scores compared to the scores attributed to the other groups, thus demonstrating the negative responses by the testers of these variables. When correlated with the other attributes, this group was in contrast to the overall appearance (GP), which means that there was a negative correlation between them; that is, there was a negative association according to the tasters between the overall appearance. However, as higher grades were attributed to flavor and aftertaste, lower grades were attributed to overall appearance. There was a positive correlation between the groups formed by the attributes of butter/rancid odor (OB), aromatic odor (OA), goat milk odor (OG) and overall odor intensity (OI) because this group was in the positive quadrant, demonstrating the acceptance by the tasters of these variables. However, as these characteristics moved away from the abscissa, they were less correlated with overall assessment.

## 4. Discussion

Moura et al. [3] observed that different levels of total dissolved solids (640, 3188; 5740; and 8326 mg/L) in the water fed to sheep do not cause changes in their ingestive behavior. Water up to 8326 mg TDS/ l can be an alternative strategic and seasonal use for watering crossbred sheep Santa Ines created in the semiarid region of Northeast Brazil [3]. In another study with Nguni goats, Mpendulo et al. [22] observed that they tolerated the highest water salinity levels tested, 11 g L^−1^. However, Mdletshe et al. [23], studying the effects of saline water on the physiological responses of lactating Nguni goats, observed that 11 g L^−1^ of salt in the water negatively affected water consumption, weight gain, feed intake and physiological (respiratory and heart rate) characteristics. According to Cardoso et al. [11] the levels of total solids ranging from 640 to 9600 mg L^−1^ in water for goats do not cause changes in their physiological variables, ingestive behavior, and efficiency. Therefore, water with up to 9600 mg TDS L^−1^ can be used strategically in the desedentation of goats in semi-arid regions.

In studies with lactating goats consuming water with different levels of salinity (640, 3188, 5740, and 8326 mg L^‒1^ total dissolved solids (TDS)), the authors observed that the salinity has no effect on animal nutrient intake, digestibility, or even milk yield [24]. However, increasing the water salinity leads to higher water intakes [24]. Similarly, in sheep, Elgharbi et al. [25] observed that the effect of saline water (10 g L^−1^) did not affect milk production and composition. However, the available findings on the effect of water salinity on milk production and composition of lactating ruminants are limited and to some extent contradictory [26], whereas Pereira et al. [27] observed a significant decreasing linear effect (*p* < 0.01) as they increased the proportion of silk flower in the diets, providing ad libitum fresh water, of lactating goats producing 1.32 kg day ^‒1^ of milk, averaging 7.06 kg/animal/day.

In the present research, an increase of 150% in the consumption of water was observed as the level of salinity in the water increased. Ingestion of high concentrations of salt induces homeostatic responses in the animal, which increases water intake [11,28,29,30,31,32]. Alves et al. [33] studied the effects of TDS levels similar to the ones used in the current study on the digestion, performance, and water balance of heifers and observed a linear increase in voluntary water consumption. The NRC [12] reported that the total water intake for small ruminants could be estimated by the following equation: Total Water Intake = Dry Matter Intake × 3.86 − 0.99. Nevertheless, this formula disregards different water salinity levels, which usually increase water intake by ruminants or the physiological status of lactating animals, which may also influence nutrient intake, elevating water intake by up to 50%. Conversely, the total water intake of goats was different than that predicted by the equation.

The increase in the value of milk acidity may be due to a probable imbalance between the minerals present in milk. The mineral composition of the milk is determined by the ionic balance between the phases in which the minerals that make up the milk are found. When there is a mineral imbalance in the milk, there will be compensation for this imbalance, and this may cause an increase in the ionic calcium, which causes a change in milk stability [34].

The increase in the levels of minerals in the milk can modify the organoleptic properties of the milk, mainly causing a more pronounced flavor of the product, and thus harming its commercialization [35]. A pronounced flavor was not observed in this work, as a favorable result was found for the sensorial characteristics of the milk of animals that were raised in regions where the means of the total dissolved solid levels of the groundwater were within the same range as those studied here. There are studies that seek to minimize the “goat taste” in the milk of this species, as well as in its derivatives [24,36,37].

## 5. Conclusions

Based on the analyses performed in this experiment, it was verified that water with different total dissolved solids (640, 3188, 5740, and 8326 mg L^−1^), when provided for short periods of up to 48 days, does not alter the production, physicochemical characteristics, or organoleptic properties of goat’s milk. Therefore, water containing these salinities can be offered to dairy goats without causing damage to the milk.

Therefore, the present study offers interesting conclusions to be applied in arid areas whose water has high salinity and offers farmers the possibility of using said water in the feeding of sheep without this diminishing the quality of the milk.

## Figures and Tables

**Table 1:** Chemical composition of the experimental diets.

Ingredient	Buffel-Grass Hay	Ground Corn	Soybean Meal	Total Diet
Dry matter (DM)	92.40	89.08	88.99	90.95
Organic matter (OM)	94.43	98.60	98.41	94.51
Mineral matter (MM)	5.57	1.40	1.59	5.47
Crude protein (CP)	6.47	10.31	49.04	13.98
Ether extract (EE)	1.94	6.17	2.21	3.34
NDFap	76.69	17.81	22.72	47.63
ADFap	49.69	4.66	10.76	27.99
Lignin	9.98	1.97	4.49	6.31
Cellulose	25.03	3.39	4.44	14.30
Hemicellulose	23.79	12.45	11.90	17.78
Total carbohydrates	86.02	82.12	47.16	77.18
NFCap	9.38	64.31	24.44	29.55
ADIP	0.96	0.94	1.36	0.99
Total digestible nutrients (TDN)	32.25 *	85.00 **	82.00 **	56.70

NDFap: neutral detergent fiber corrected for ash and protein; ADFap: acid detergent fiber corrected for ash and protein; NFCap: non-fibrous carbohydrates corrected for ash and protein; ADIP: acid detergent indigestible protein; * Valadares Filho et al. [13]; ** Moreira et al. [14].

**Table 2:** Mean values of the conductivity variables, total dissolved solids (sodium, chlorine, calcium, magnesium, and potassium), and alkalinity of the waters offered to the experimental goats.

Variables	Total Dissolved Solids in the Water (mg L^−1^)			
	640	3188	5740	8326
Conductivity (ds m^−1^)	1.00	4.98	8.97	13.01
Sodium (mg L^−1^)	253.0	1030	1840	3680
Chlorine (mg L^−1^)	632.8	1350	2800	4150
Calcium (mg L^−1^)	11.60	13.20	14.80	18.80
Magnesium (mg L^−1^)	26.40	17.16	11.04	10.32
Potassium (mg L^−1^)	5.86	5.47	3.13	5.87
Alkalinity (mg L^−1^)	13.80	14.20	14.60	24.50

**Table 3:** Descriptors used in the quantitative descriptive sensory analysis of goat milk.

Descriptor	Definition
Flavor	Mixed experience of olfactory, gustatory and tactile sensations perceived during tasting. Flavor slightly bitter with buttery perception
Odor	Organoleptic property perceived by olfactory organ when certain volatile substances are smelled
Overall appearance ^a^	Sum of the quality attributes that will contribute to determining the degree of product acceptance by panelists
Aftertaste	Salty aftertaste remaining after ingestion of the product

^a^ 0 = very bad; 5 = very good.

**Table 4:** Physicochemical composition of milk of goats of the Alpine breeds subjected to treatments with increasing levels of total dissolved solids over a period of 48 days of exposure.

Variable	Total Dissolved Solids in the Water (mg L^−1^)				RMSE ^1^	Significance	
	640	3188	5740	8326		Lin ^2^	Quad ^3^
Milk yield (g day^−^¹)	1.79	1.85	1.76	1.86	0.18	0.582	0.902
WIB (kg day^−1^)	6.08	6.98	7.82	9.11	1.33	0.001	0.725
TDE (%)	10.40	10.46	9.98	10.20	0.60	0.277	0.180
DDE (%)	7.68	7.81	7.46	7.66	0.42	0.792	0.346
Moisture	89.60	89.54	90.02	89.80	0.60	0.277	0.180
Ash (%)	0.73 *	0.76	0.68	0.72	0.08	0.548	0.112
Fat (%)	2.72	2.65	2.51	2.54	0.34	0.098	0.209
Protein (%)	3.45 *	3.67	3.15	3.49	0.31	0.916	0.146
Lactose	4.75	4.70	4.75	4.54	0.27	0.215	0.064
Acidity	0.12 *	0.14	0.13	0.14	0.02	0.024	0.321
Density (g/cm^3^)	1031.10	1031.70	1031.00	1031.30	1.01	0.529	0.325

WIB = water intake from bucket; ^1^ root mean standard error; ^2^ significance for linear effect; ^3^ significance for quadratic effect; * = orthogonal contrast: control versus water salinity levels (*p* < 0.05).

**Table 5:** Mineral composition of the milk of goats of the Alpine breed subjected to treatments with increasing levels of total dissolved solids over a period of 48 days of exposure.

Variable	Total Dissolved Solids in the Water (mg L^−1^)				RMSE ¹	Significance	
	640	3188	5740	8326		Lin ^2^	Quad ^3^
Phosphorous	104.47	103.90	102.86	111.77	18.48	0.285	0.279
Chlorine	245.18	256.68	251.37	248.27	12.99	0.622	0.062
Calcium	129.41	130.32	133.39	123.61	15.42	0.382	0.145
Sodium	158.86	164.43	162.77	160.62	8.50	0.698	0.057
Potassium	234.28	235.55	239.46	231.65	31.51	0.902	0.539

^1^ root standard mean error; ^2^ significance for linear effect; ^3^ significance for quadratic effect.

**Table 6:** Mean scores of the quantitative descriptive analysis of the milk of goats of the Alpine breed subjected to treatments with increasing levels of total dissolved solids over a period of 48 days of exposure.

Variable	Total Dissolved Solids in the Water (mg L^−1^)				RMSE ¹	Significance	
	640	3188	5740	8326		Lin ^2^	Quad ^3^
Odor							
Overall intensity	2.39	2.12	2.27	2.30	1.26	0.519	0.560
Goat milk	2.27	1.85	2.00	2.06	1.25	0.224	0.494
Butter/rancid	1.91	1.61	1.73	1.73	0.96	0.247	0.613
Aromatic	2.12	1.73	1.91	2.00	1.19	0.307	0.355
Flavor							
Overall intensity	2.85	2.73	2.58	2.52	1.14	0.289	0.444
Goat Milk	2.76	3.09	2.64	2.55	1.27	0.991	0.083
Butter/rancid	2.36	2.15	2.30	2.03	1.00	0.320	0.620
Aromatic	2.42	2.48	2.39	2.15	1.21	0.746	0.264
Aftertaste							
Intensity	2.55	2.85	2.52	2.30	1.20	0.957	0.066
Persistence	2.33	2.58	2.48	2.15	1.22	0.766	0.159
Global Appearance	3.06	3.37	3.18	3.48	0.95	0.144	0.607

^1^ root standard mean error; ^2^ significance for linear effect; ^3^ significance for quadratic effect.

## Data Availability

All data obtained during this research are presented in the manuscript.
